# Association of *BDNF *Gene Polymorphism With Asthma in Polish Children

**DOI:** 10.1097/WOX.0b013e3181eedb68

**Published:** 2010-09-15

**Authors:** Aleksandra Szczepankiewicz, Anna Bręborowicz, Paulina Sobkowiak, Anna Popiel

**Affiliations:** 1 Laboratory of Cellular and Molecular Analysis, Department of Pediatric Pulmonology, Allergy and Clinical Immunology, IIIrd Department of Pediatrics, Poznan University of Medical Sciences, 27/33 Szpitalna St., 60-572 Poznan, Poland; 2 Department of Pediatric Pulmonology, Allergy and Clinical Immunology, IIIrd Department of Pediatrics, Poznan University of Medical Sciences, Poland

**Keywords:** asthma, BDNF gene, polymorphism

## Abstract

Allergic asthma is associated with changes in neuronal control in the airways that modulate inflammation and airway hyperresponsiveness. The link between inflammation and neuronal dysfunction is provided mainly by neurotrophins, in particular Brain Derived Neurotrophic Factor (BDNF). In humans, significantly higher serum BDNF levels have been observed in asthmatic patients when compared with healthy subjects. BDNF levels are also significantly higher in untreated asthmatic patients in comparison to those treated with inhaled glucocorticoids and nonasthmatic controls. Allergic inflammation increases local BDNF production and its concentration correlates with clinical parameters of allergic airway dysfunction. The aim of this study was to analyze the possible association of BDNF gene polymorphism with susceptibility to asthma and disease severity. We analyzed 146 children diagnosed with asthma and 227 children from the control group. Genotyping of 4 *BDNF *polymorphisms (rs12273363, rs7124442, rs6265, and rs2030324) was done with use of PCR-RFLP and TaqMan SNP genotyping assay. Genetic association analysis was performed in Statistica. Linkage disequilibrium was determined with Haploview. Single marker analysis revealed a significant association of C allele of rs2030324 polymorphism with asthma susceptibility (*P *= 0.048). However, *BDNF *polymorphism was not associated with severe asthma. Strong linkage disequilibrium was observed between all of the *BDNF *polymorphisms analyzed grouped in one haplotype block. We found a significant association of TTGC haplotype with asthma (*P *= 0.025). Our results suggest that genetic variation in the *BDNF *gene may contribute to asthma susceptibility in case of rs2030324 polymorphism and TTGC haplotype, however it does not influence asthma severity.

## Introduction

Asthma is the most common chronic disease of childhood and is characterized by the reversible airflow obstruction with chronic inflammation of the airways. Despite allergic inflammation, dysregulation of lung neuronal network takes place in asthma. Particularly important role in neuro-immunologic interactions play neurotrophins that are able to regulate allergic inflammation. Brain-derived neurotrophic factor (BDNF) is one of key neurotrophins involved in the regulation of differentiation and survival of various types of neurons (including sensory neurons) and promotion of eosinophils survival.

Increased BDNF levels have been found in bronchoalveolar lavage fluid after allergen challenge in allergic asthmatic patients[1,2] and BDNF levels are significantly higher in untreated asthmatics in comparison to patients treated with inhaled glucocorticoids and nonasthmatic controls [3]. Furthermore, allergic inflammation increases local BDNF production and enhanced BDNF concentration correlates with clinical parameters of allergic airway dysfunction [4]. The specificity of BDNF to neuronal dysfunction in allergic inflammation is supported by the finding that the reduction of serum BDNF levels after inhaled glucocorticoid therapy is not correlated with changes in acetylcholine responsiveness of the airways [3]. Recent report that dendritic cells may be activated by neurotrophins such as BDNF or NGF suggests their role in regulation of allergic sensitization and inflammation [5]. However, neurotrophins may also act at levels different from allergic inflammation, mediating in nonspecific airway responsiveness.

Given the biologic role of BDNF and conflicting results from previous association studies, we assumed to investigate further if the variation in the BDNF gene may be relevant for asthma susceptibility and severity in Polish population.

## Subjects and Method

### Patients

The study was performed on Polish sample of 146 asthmatic patients of white origin in age from 6 to 18 years old (86 boys with a mean age of 11.5 years, SD = 3.5; 60 girls with a mean age of 11.7 years, SD = 3.7). Patients were recruited from inpatients from Wielkopolska region, considered as ethnically homogenous,[6] and were treated for asthma in the Department of Pediatric Pulmonology, Allergy and Clinical Immunology of Poznan University of Medical Sciences. Asthma diagnosis was made according to GINA recommendation, based on clinical asthma symptoms and lung function test (bronchodilator responsiveness, exercise induced hyperresponsiveness); bronchodilator response was assessed 20 minutes after administration of 200 mcg of Salbutamol MDI via a holding chamber (Volumatic) and a ≥ 12% increase in FEV_1 _was diagnostic; bronchial hyperresponsiveness was assessed by exercise test using a 6 minute run on the treadmill and a postexercise fall in FEV_1 _of ≥ 15% was considered positive.

Severe asthma was defined as follows: symptoms requiring daily therapy with high-dose inhaled corticosteroids (>800 mcg budesonide or >500 mcg fluticasone), despite regular therapy with long acting *β*_2_-agonists and/or leukotriene antagonist and/or theophylline (slow releasing), 1 or more emergency care visit or oral steroids bursts per year. In our group, 55 children met the criteria for severe asthma.

Clinical diagnosis of atopy depended on current or past symptoms of atopic dermatitis, allergic rhinoconjunctivitis (seasonal or perennial), or food allergy. Atopy was confirmed in 105 children (72%) that fulfilled one of the after criteria: total IgE level higher than the upper normal limits for age; positive skin prick test to at least one aero-allergen (Dermatophagoides pteronyssinus, Dermatophagoides farinae, cat, dog, feathers, Alternaria alternata, Cladosporium herbarum; pollen: grass mix, rye, birch pollen, alder, hazel; Allergopharma, Germany). Any reaction with mean wheal diameter at least 3 mm greater than negative control was regarded positive and defined atopy [7]. Total serum IgE level was measured by a fluoroimmunossay with Pharmacia UniCap 100 System (Pharmacia, Uppsala, Sweden) following manufacturer's instruction. The upper limits of normal range for total IgE was age-dependent (70 kU/l for 6 years children; 79 KU/L for 7 years children, 89 KU/L for 8 years children, 98 KU/L for 9 years children, 107.0 KU/L for children of 10 years and older).

### Control Group

Control group consisted of 227 healthy subjects of white origin (111 boys with a mean age of 11.5 years, SD = 3.0; 116 girls with a mean age of 12.4 years, SD = 3.6). Control subjects were also recruited from the same geographic region (Wielkopolska) from the group of carefully chosen volunteers without asthma and allergy symptoms. Any allergic diseases or asthma were excluded based on clinical examination, history, spirometry, and exhaled NO measurement.

All participants and their parents have given written informed consent. Local ethics committee accepted the project. Study was performed in compliance with the Code of Ethics of the World Medical Association (Declaration of Helsinki).

### Genotyping

Four SNPs were chosen for analysis in this study; rs6265 (Val66Met) that has been previously shown to alter the intracellular trafficking and packaging of pro-BDNF[8]; and 3 SNPs: rs2030324 (intronic) and rs12273363 (5'UTR), and rs7124442 (3'UTR) that were identified as tag SNPs for the *BDNF *gene region utilizing HapMap Caucasian data (http://www.hapmap.org/).

The DNA was extracted from 10 mL of EDTA anticoagulated whole blood using the salting out method [9]. Genotyping of 4 *BDNF *polymorphisms (rs12273363, rs7124442, rs6265, and rs2030324) was done with use of PCR-RFLP and TaqMan SNP genotyping assay C_27833027_10 (Applied Biosystems). The sequences of the primers and conditions of PCR-RFLP analysis for the 3 polymorphisms (rs12273363, rs6265, and rs2030324) were shown in Table 1. The amplification for TaqMan SNP genotyping assay plates was done in ABI PRISM 7900HT Sequence Detection System. Data acquisition and analysis was performed using the allelic discrimination analysis module in SDS v2.1 software (Applied Biosystems).

**Table 1:** Characterization of Reaction Conditions for the Analyzed SNPs

SNP	Primers	PCR Product (bp)	Tm (°C)	Restriction Enzyme	Allele Sizes (bp)	Source
Rs12273363	5'-AGGCACAGCGATGCTGCAGAGGA-3'	200	55	BseGI	C: 34, 166	Own design
	5' CCTTGAAGCAGCCACTGAATG-3'				T: 34, 34, 132	
Rs6265	5'-ACTCTGGAGAGCGTGAATGG-3'	197	57	Eco72I	A: 197	Own design
	5' AGAAGAGGAGGCTCCAAAGG-3'				G: 125, 72	
Rs2030324	5' TTGCACATCCTGCTCAAGTC-3'	348	55	TaiI	T: 348	Own design
	5'-TTGCTAGGAGAAAAGCCATGA				C: 266, 82	
Rs7124442	TaqMan genotyping assay C_27833027_10	--	60	--	--	Applied Biosystems

The uncut PCR products for *BDNF *polymorphisms analyzed with PCR-RFLP (rs2030324 and rs6265) were digested twice to confirm the results. For each reaction plate genomic control DNA samples and nontemplate controls (water) were included. The control of RFLP analysis and TaqMan SNP genotyping assay was also performed (15% of randomly chosen samples from both groups) to check for genotyping accuracy. The genotyping was performed without knowing the clinical outcome of the patient.

### Statistical Analysis

The Pearson's *χ*^2 ^test and Fisher exact test were used to test differences in the genotypic and allelic, respectively, distribution in case control. Calculations were performed using the STATISTICA version 7.1 software. Odds ratios (ORs) with a 95% confidence interval (CI) were calculated using demo of GraphPad InStat 3 program. Concordance with Hardy-Weinberg law was performed using "Utility Programs For Analysis Of Genetic Linkage" application (J. Ott, 1988). We also performed linkage disequilibrium analysis of the analyzed polymorphisms of *BDNF *gene using free online software Haploview version 4.1 from the Website http://www.broad.mit.edu/mpg/haplview/index.php[10]. Power calculations were done in Quanto v.1.2.3 with OR values between 1.0 and 1.4 and for two-sided associations were as follows: for rs12273363 - 20%, for rs2030324 - 49%, for rs6265 - 34.6%, and for rs7124442 - 42%.

## Results

### Hardy-Weinberg Analysis

Genotype distributions for all studied polymorphisms in the *BDNF *gene were in concordance with Hardy-Weinberg law in both cases and control subjects (*P *> 0.05).

### Genotyping and Allele Frequencies

Minor allele frequencies for each *BDNF *polymorphism were as follows: rs2030324 C = 0.469; rs7124442 T = 0.248; rs12273363 T = 0.149; and rs6265 G = 0.177. The genotyping success rates were between 94.5-98.6%. Genotyping error rates for all the polymorphisms were <1%.

### Genetic Association Analysis

We found C allele of rs2030324 polymorphism to be significantly associated with asthma (*P *= 0.048). In analysis stratified by sex this polymorphism was also associated with asthma in boys (*P *= 0.009) but not in girls (*P *= 0.699). In analysis of *BDNF *polymorphisms with asthma severity we did not find any significant association (Table 2).

**Table 2:** Genotype Distributions and Allele Frequencies of 4 *BDNF *Polymorphisms for Asthmatic Patients Versus Control Group (Figures in Parentheses Indicate Percentages)

Polymorphism	n	Asthma	n	Control	*P *Value	OR with 95%CI
Rs12273363						
Genotypes						
CC	144	4 (2.78)	216	5 (2.31)	0.373	1.25 0.81-1.92
CT		30 (20.83)		59 (27.31)		
TT		110 (76.39)		152 (70.37)		
Alelles						
C	288	38 (13.20)	432	69 (15.98)	0.336	
T		250 (86.80)		363 (84.02)		
Rs6265						
Genotypes						
AA	146	3 (2.05)	218	9 (4.13)	0.224	
AG		37 (25.34)		68 (31.19)		1.42 0.95-2.12
GG		106 (72.60)		141 (64.68)		
Alelles						
A	292	43 (14.73)	436	86 (19.72)	0.092	
G		249 (85.27)		350 (80.28)		
Rs2030324						
Genotypes						
TT	146	31 (21.23)	214	53 (24.77)	0.061	1.35 1.00-1.82
CT		62 (42.47)		108 (50.47)		
CC		53 (36.30)		53 (24.77)		
Alelles						
T	292	124 (42.50)	428	214 (50.00)	0.048*	
C		168 (57.50)		214 (50.00)		
Rs7124442						
Genotypes						
CC	131	79 (60.31)	227	126 (55.51)	0.354	0.93 0.68-1.32
CT		41 (31.30)		87 (38.33)		
TT		11 (8.40)		14 (6.17)		
Alelles						
C	262	199 (75.96)	454	339 (74.67)	0.720	
T		63 (24.04)		115 (25.33)		

*Indicates significance.

### Linkage Disequilibrium and Haplotype Analysis

**Figure 1:**
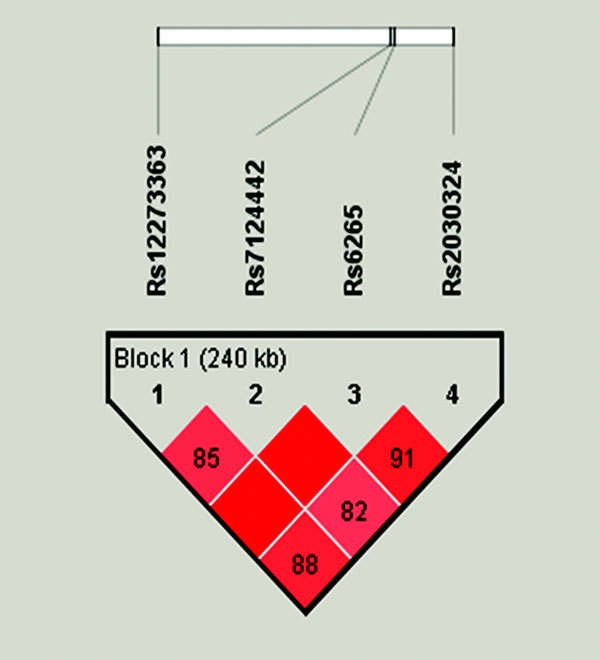
**Relative positions and LD estimates between 4 *BDNF *polymorphisms in the analyzed population**. Coloured squares correspond to D' values with numerical estimates given within the squares.

Moderate LD was observed between all of the *BDNF *polymorphisms analyzed. Haploview analysis defined one block for all the SNPs analyzed (Figure 1). We observed 7 common haplotypes (estimated population frequency >0.005). Haplotype analysis for relationship with affection status using Haploview found a significant association of TTGC haplotype with asthma (Table 3). However, after adjusting significance level for multiple comparisons using 10,000 permutations, we have found only marginal association of this haplotype with asthma (*P *= 0.07).

**Table 3:** Results of Haploview Haplotype Analysis Between Asthmatic Patients and Healthy Control Subjects in the Analyzed Population

Haplotype	Frequency	Case: Control Ratio	***χ***^**2**^	*P *Value
TTGC	0.494	0.545, 0.461	5.013	0.025*
TTAT	0.165	0.140, 0.182	2.362	0.124
CCGT	0.124	0.118, 0.129	0.182	0.670
TCGT	0.099	0.097, 0.101	0.035	0.852
TTGT	0.067	0.057, 0.074	0.793	0.373
TCGC	0.018	0.024, 0.013	1.184	0.276
CTGT	0.011	0.012, 0.011	0.023	0.878

*Indicates significance.

## Discussion

The main finding of our study is an association of rs2030324 *BDNF *polymorphism with asthma in our pediatric population. This association was also observed for the male sex. No association was found for the other studied polymorphisms.

Previous studies investigating the impact of genetic variation in BDNF have shown conflicting results [7,11,12]. Negative association results presented here for 3 BDNF polymorphisms (rs12273363, rs7124442, rs6265) are consistent with those obtained in a German cohort[11] and also with data published for a large British cohort of asthmatic siblings [12]. However, the association observed for rs2030324 polymorphism was not observed previously [12]. It is difficult to discuss our results as there are no other studies available for asthma that could verify our results. The recent study by Zeilinger et al[11] reported no association of BDNF gene with asthma or any atopic disease in a large pediatric ISAAC cohort. However, the associated rs2030324 polymorphism was not among those SNPs analyzed by this group.

In linkage disequilibrium analysis we observed moderate linkage between 4 analyzed *BDNF *polymorphisms located in one LD block. In this block we found that TTGC haplotype was associated with higher risk of developing asthma in our population. Such an association was not found previously [12]. This may result not only from the differences in sample size, but also from ethnic disparities between the Polish and British samples.

The main limitation of our study is a relatively small sample size in comparison to the other studies reporting on the role of BDNF polymorphism in asthma. However, our group of patients and controls is carefully characterized with doctor's diagnosis (or asthma exclusion in controls) confirmed by lung function tests and clinical markers of allergic inflammation (skin, prick test, elevated IgE level, comorbid atopic diseases).
